# Supplementary material for: Long-term surveillance defines spatial and temporal patterns implicating Culex tarsalis as the primary vector of West Nile virus
Source: Sci Rep. 2019 Apr 29;9:6637. doi: 10.1038/s41598-019-43246-y (PMC6488619; doi:10.1038/s41598-019-43246-y)
Supplement: Supplementary file 1 — Supplemental information [file 41598_2019_43246_MOESM1_ESM.pdf]

Supplemental Data

Long-term surveillance defines spatial and temporal patterns implicating *Culex tarsalis* as the primary vector of West Nile virus

Brendan M. Dunphy[1], Kristofer B. Kovach[1], Ella J. Gehrke[1], Eleanor N. Field[1], Wayne A. Rowley[1], Lyric C. Bartholomay[2], and Ryan C. Smith[1*]

[1] Department of Entomology, Iowa State University, Ames, Iowa, 50011, USA
[2] Department of Pathobiological Sciences, University of Wisconsin, Madison, WI 53706

*Correspondence: smithr@iastate.edu

Supplemental Information

Supplemental Tables
Table S1. Summary of combined WNV+ pools for *Culex* species in Iowa, 2002-2016.

Supplemental Figures
Figure S1. Identification of *Culex pipiens* group blood meals by species.

Species	# Specimens tested	# Pools tested	# pools WNV+	MIR
Cx. erraticus	4585	283	6	1.31
Cx. pipiens group	129763	4888	241	1.86
Cx. tarsalis	22863	731	46	2.01

Table S1. Summary of combined WNV+ pools for *Culex* species in Iowa, 2002-2016. Mosquito number, pools tested, and WNV+ pools for each *Culex* species in Iowa. Minimum infection rate (MIR) was calculated as a bias-corrected maximum likelihood estimate.

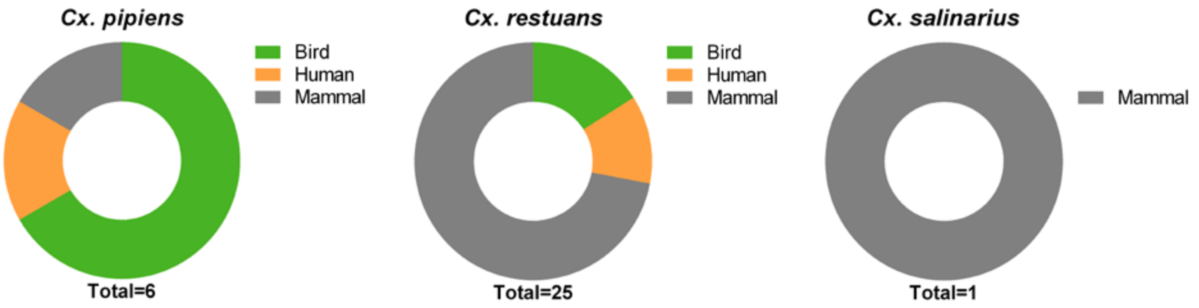

Figure S1. Identification of *Culex pipiens* group blood meals by species.

Culex pipiens group mosquito samples in which the blood meal source was identified were further speciated to confirm if the sample was *Cx. pipiens*, *Cx. restuans*, or *Cx. salinarius*. The percentage of blood meals taken respectively from birds, humans, and non-human mammals are displayed for each mosquito species. The total number (n) of identified blood meals is displayed below.
